# Prevalence of Pulmonary Tuberculosis among Prison Inmates at Mbarara Central Prison, South Western Uganda

**DOI:** 10.9734/AIR/2014/10676

**Published:** 2014-06-25

**Authors:** Judith Owokuhaisa, Eric Thokerunga, Joel Bazira

**Affiliations:** 1 Department of Nursing, Faculty of Medicine, Mbarara University of Science and Technology, Mbarara, Uganda; 2 Department of Medical Laboratory Sciences, Faculty of Medicine, Mbarara University of Science and Technology, Mbarara, Uganda; 3 Department of Microbiology, Mbarara University of Science and Technology, Mbarara, Uganda

**Keywords:** Tuberculosis, prison inmates, prevalence, Mbarara central prison

## Abstract

**Aims:**

This study was conducted to determine the prevalence of tuberculosis among prison inmates at Mbarara Central prison

**Design:**

A cross sectional study was carried out at Mbarara Central Prison in Mbarara district, Kiswahili cell in Mbarara municipality among female and male prison in mates between June 2012 to August 2012. A questionnaire was administered to each prison inmate who consented in writing and two sputum specimens were collected and examined by Ziehl-Neelsen technique.

**Results:**

At the time of the study, the prison had 900 inmates (both males and females). A total of 648 in mates were screened and 248 inmates enrolled in the study. Of the 248 inmates, 5 inmates were new cases of TB while 29 inmates were already on TB treatment. The median age of participants was 28 years (23.5-33 IQR) and 96.4% were males with majority (22.6%) coming from Mbarara as a home district. The participants had stayed in prison for a median duration of 2 years (1-3 IQR) and 23.7% had ever been in prison before. The median number of inmates per cell was 140 (138-149 IQR) and inmates (female and male) had a body mass index of 21.4 (19.9-22.6 IQR) and 20.2 (19.2-26.7 IQR) respectively. Of the inmates evaluated, 68.8% reported cough for 2 or more weeks. Other symptoms reported were weight loss (in 40.7%) and night sweats (in 35.8%). Of the 248 inmates evaluated, 95 inmates were tested for HIV and 4.1% were HIV serology positive.

**Conclusion:**

The prevalence of TB in Mbarara Central prison South Western Uganda is low but calls for continued surveillance through regular TB screening.

## 1. INTRODUCTION

Uganda ranks 16^th^ on the list of the 22 most tuberculosis burdened countries in the world. It has an estimated national tuberculosis prevalence of 651/100 000 [1] but this might have been underestimated as the national notification data is often incomplete due to inadequate reporting and recordings. More so, the national tuberculosis control programme recognizes passive case detection as the strategy for diagnosis which involves only people with signs and symptoms voluntarily going to the health care units for diagnosis thus leaving a majority of people with poor health seeking behaviors undiagnosed and continuing to spread the disease.

Since tuberculosis is an air borne disease, overcrowding as is common in prisons creates prime conditions for its spread [2]. More so, a lifestyle, such as tobacco/cigarette smoking which is also common in prisons could increase the chances of developing clinical TB four-fold [3], due to the various effects of smoking on components of both innate and adaptive immunity.

Prison inmates form a group of people at high risk of tuberculosis infection. Studies show that the prevalence of TB among prison inmates is 6 – 10 times higher than in the general population [4]. Tiny ventilations and crowded cells are the nationwide characteristics of Uganda prison cells creating favorable conditions for TB spread among the prison inmates [5]. Epidemiologic studies have shown that risk of TB increases with close contacts of sputum-smear-positive patients and that the prevalence of clinical disease among intimate contacts of TB cases is high [6]. Similarly, there are growing fears that the high rate of TB in prisons coupled with weak health care systems are contributing to the emergence of Multi-Drug Resistant Tuberculosis (MDRTB) strains [7]. This study therefore, sought to find out the prevalence of tuberculosis among prison inmates at Mbarara central prison, Kiswahili cell Mbarara District, South Western Uganda.

## 2. MATERIALS AND METHODS

### 2.1 Study Design, Setting and Population

A cross-sectional survey was carried out among prison inmates at Mbarara Central Prison located in Kiswahili cell Mbarara Municipality 100 meters away from Bank of Uganda along the Mbarara-Kabale highway. The study population comprised of both male and female prison inmates who consented in writing to participate in the study.

### 2.2 Data Collection

After getting signed informed consent from the prison inmates, we administered questionnaires to them face to face collecting data on socio-demographic characteristics, presence of chronic cough and TB risk factors like smoking, malnutrition (measured body mass index) and HIV infection. Inmates who admitted to not having tested for HIV and were willing to test were all screened for the disease. Sputum samples were collected from inmates who were found with signs and symptoms suggestive of TB disease and were requested to give 2 sputum specimens; 1 on spot, and the second early morning sample which were collected in plastic, wide mouthed, leak proof containers labeled with the respondents’ unique code and then transported in cool boxes to Mbarara University department of Microbiology for microscopy.

### 2.3 Laboratory Procedures

Smear microscopy was done on every sample collected by experienced TB laboratory technologists using the conventional Ziehl-Nielsen technique with positive smears quantified using the International Union against TB and Lung Disease standard ^(19, 1)^. Known negative and positive smears were stained alongside the test samples as controls. No sputum culture was performed.

### 2.4 Nutritional Assessment

Body weight was determined to the nearest 0.1 kg on an electronic scale and height was measured to the nearest 0.1 cm. Body mass index (BMI) defined as the weight in kilogram of the individual divided by the square of the height in meter, was used to determine the nutritional status of the inmates into severe malnutrition (BMI<15.9 kg/m^2^), moderate malnutrition (BMI=16-16.9 kg/m^2^), mild malnutrition (BMI=17-18.4 kg/m^2^) and normal BMI=18.5-25 kg/m^2^ as recommended by WHO^(21)^.

### 2.5 Statistical Analysis

The data was coded and entered in a computer and analyzed using the statistical package for social sciences (SPSS) version 20. Data was presented and described in tables.

### 2.6 Ethical Approval

The study was approved by Mbarara University of Science and Technology Institution Research Committee (MUST IRC), and the Prisons Administration gave us permission to carry out the study. The participants were told their participation was on a voluntary basis and no incentives to participate were provided. The potential benefits for participation included testing for tuberculosis and referral for treatment in case their tests were positive.

## 3. RESULTS

The total number of inmates in the prison during the study period was 900 of which 677 were screened and 223 not screened (had not consented). Of the 677 inmates screened, 248 met the inclusion criteria and submitted spot and early morning sputum, while 429 inmates did not meet the inclusion criteria.

**Fig. 1:**
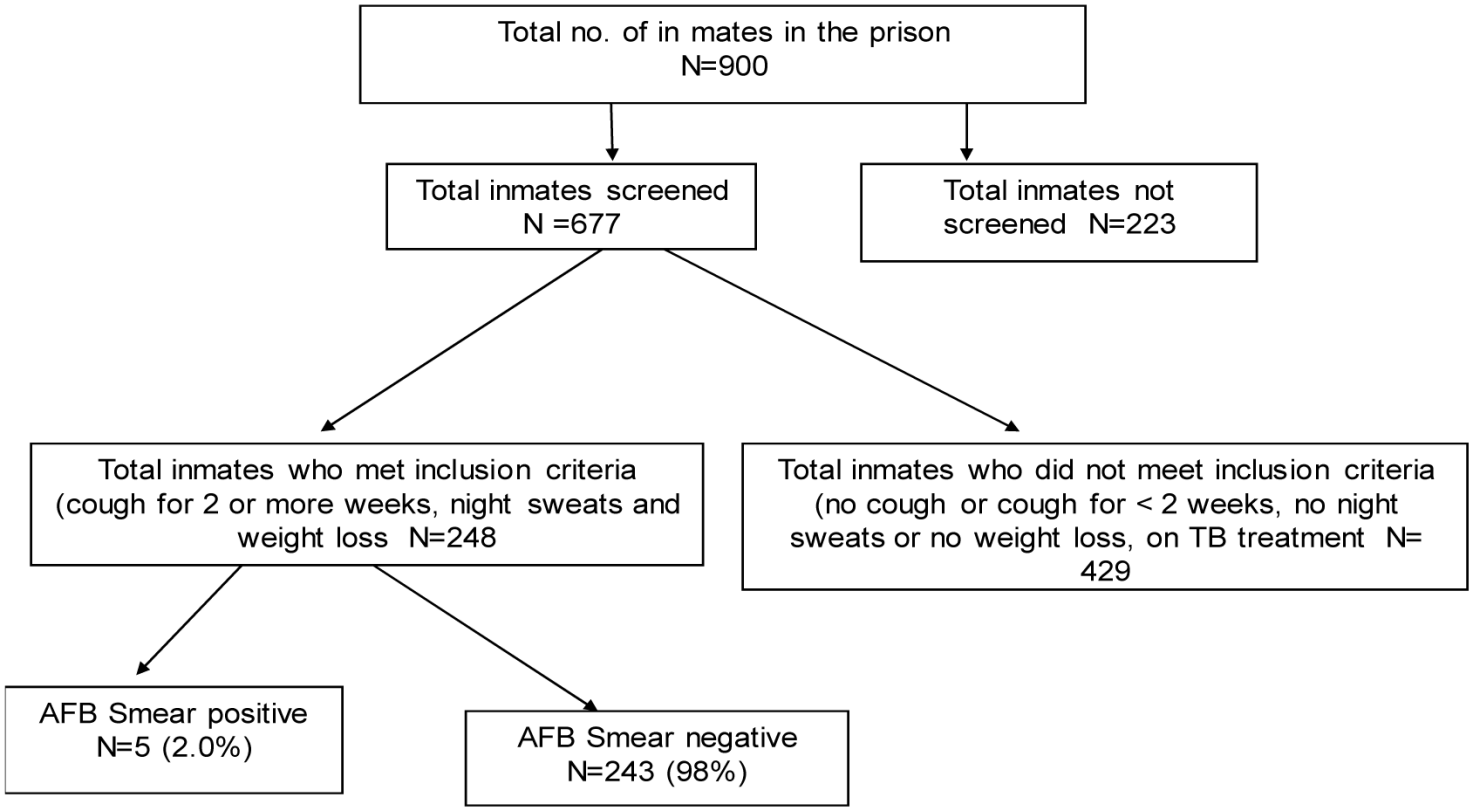
Study profile

As shown in Fig. 1, five (2.0%) out of the 248 inmates whose sputum samples were collected for microscopy, were diagnosed with smear positive tuberculosis.

Three quarters of the study participants were males (87.3%) with a median age of 27 (22 – 32). Majority were inhabitants of Mbarara district (21.5%). Median duration in prison was 2 years (inter quartile [IQR] 1-3) with majority having never been in prison before (76%). Median number of people per ward/cell was 140 (IQR 138-149). Median body mass index of the symptomatic participants (both females and males) was 21.4 (IQR 19.9-22.6) and 20.2 (IQR 19.2-26.7) respectively. Fifty four percent of participants reported to have ever smoked and majority about 80% were currently smoking and 72% admitted to have ever drunk alcohol. HIV test results showed 4.1% positivity among inmates who met the inclusion criteria in the study.

All the positive sputum smears were from male inmates, with majority in the age range of 20-30 years. All the in mates who were smear positive had a normal BMI except one who had a low BMI of 17.51 (Table 2).

## 4. DISCUSSION

The prevalence of PTB among inmates in Mbarara Central prison during the study period was 2.0% which correlates with studies among prison inmates in a Nigerian medium security prison which demonstrated a PTB prevalence of 2.4% [8]. More so, another study carried out among inmates in a prison hospital in Bahia, Brazil revealed a prevalence of 2.5%which is quite similar to that found in our study (2.0%) [9], although studies from elsewhere in similar settings like Zambia and Botswana showed much higher prevalence [10,11].

The cells in the study were poorly ventilated and housed hundreds of inmates (median no. of inmates per cell was 140) who mix during day to day activities all day long from other cells in enclosed spaces. This is similar to study findings from North West Ethiopian prison which revealed that the mean number of inmates per cell was 333 [12]. This shows that overcrowding is a factor that puts inmates at risk of acquiring TB. Also given the fact that the inmates in this study were spending quite a long time in prison (Median duration of 2 years); this could have been rendering the prison to serve as a reservoir of TB transmission.

During the study HIV testing was offered to all inmates who consented to the test after counseling. Acceptance to be tested was high (Table 1) especially among in mates who did not meet the criteria for microscopic examination of sputum samples and this shows that they were eager to know of their HIV serology status.

More so, the current study revealed a total of 7 (4.1%) inmates who were found to be reactive for the HIV antibody test (see Table 1). These findings are slightly similar to the study carried out among inmates in North West Ethiopia which showed a total of 19 (7.6%) in mates with HIV infection. This reveals that HIV infection and the associated immune suppression is a major risk factor for the development of active TB in those who develop new M. tuberculosis or have latent M. tuberculosis infection.

In addition, the prevalence of TB in prisons is also often related to prison – associated risk factors as malnutrition [13,14]. In the current study, based on the BMI of prison inmates, all the inmates screened were well nourished with a median BMI of 21.4 kg/m^2^ (IQR 19.9-22.6) in females and 20.4Kg/m^2^ (IQR 19.2-26.7) among male inmates. This is in contrary of findings in a study carried out in North West Ethiopian prison inmates where 46.2% of the TB positive inmates were under nourished (BMI <18.5kg/m^2^) [12]. Findings from this study show that inmates were not under nourished despite the long duration spent in the prison probably they had adjusted to the new environment or the feeding while in the prison was favorable to help them maintain good nutrition status.

The development of TB observed in inmates in Mbarara Central prison may also be linked to other adverse conditions like overcrowding, smoking and prolonged exposure to infective but untreated patients (Tables 1 and 2), [3], such factors are seen as independent risk factors to development of TB disease but can be synergistic [15,16].

Absence of inmates due to labour inside and outside the prison, appearance before court and refusal to be screened (223, no consent), did not allow all the inmates to be screened. Only symptomatic inmates with prolonged productive cough (2 or more weeks) were eligible for screening and only those with microscopically confirmed PTB were taken as TB cases.

However the prevalence observed in this study may be under estimation of the actual prevalence due to logistic reasons like lack of chest X-rays and culture and sensitivity facilities where asymptomatic as well as sputum negative cases were missed. The prevalence observed in this study may have been under estimated due to the ZN technique used whose sensitivity is not 100% and excludes extra pulmonary TB that require X-ray for detection which was not done in this study.

## 5. CONCLUSION

The prevalence of TB in Mbarara Central prison South Western Uganda is low but this calls for continuous screening of TB among the inmates in order to prevent increase of TB in the prison.

## Figures and Tables

**Table 1:** Description of study participants who met the inclusion criteria

**Characteristics**	**Inmates included in the study (n=248)**
	**N (%)**
Age (Years)* Median(IQR)	28 (23.5-33)
**Gender**	
Female	9 (3.6)
Male	239 (96.4)
**Home district**	
Mbarara	56 (22.6)
Isingiro	42 (17.0)
Ntungamo	29 (11.7)
Bushenyi	24 (9.7)
Ibanda	21 (8.5)
Others ##	76 (30.6)
**Duration in prison (years)**	2 (1-3)
**Ever been in prison before**	
Yes	58 (23.7)
No	187 (76.3)
Number of people per ward/ cell*	
	140 (138-149)
Body mass index*	
Female	21.4 (19.9-22.6)
Male	20.2 (19.2-26.7)
**Ever smoked**	
Yes	134 (54.0)
No	114 (46.0)
**Currently smoking**	
Yes	47 (19.7)
No	192 (80.3)
**Ever drank alcohol**	
Yes	179 (72.2)
No	69 (27.8)
**HIV test results positive**	7 (4.1)
Negative	88 (51.8)
Did not want an HIV test	75 (44.1)

* Median (Inter quartile Range [IQR]), ## others (Kabale, Masaka, Kisoro and Rwanda)

**Table 2:** Characteristics of smear positive in mates (N= 05)

In mate number	Sputum 1	Sputum 2	HIV results	BMI	Gender	Age Range (years)	Currently drinking	Currently smoking
151	Positive	Negative	Negative	20.31	Male	30-40	No	No
373	Positive	Negative	Positive	27.78	Male	30-40	Yes	No
411	Positive	Negative	Not tested	17.51	Male	30-40	Yes	No
314	Negative	Positive	Not tested	18.93	Male	30-40	No	Yes
320	Negative	Positive	Positive	22.09	Male	20-30	No	No
